# The Action of Hydrochlorate of Hyoscine

**Published:** 1887-08

**Authors:** 


### ON THE ACTION OF HYDROCHLORATE OF HYOSCINE.

UNDER the direction of Kobert, Sohrt has made a series of experiments on the physiological action and therapeutic use of hyoscine, the results of which are published in the above paper. The conclusions arrived at by Wood, as to the manner in which hyoscine influences the various organs, are to a large extent confirmed, but on some points the views of the American and German observers are not in accord.

In healthy men, the injection of one-seventieth of a grain gave rise, according to the latter, to disturbances of co-ordination and some muscular weakness; the head felt heavy, sank on the shoulder, and could only be lifted up with difficulty; the limbs likewise felt heavy, and the upper eyelid could hardly be raised; there was a sense of fullness of the head, the pupils were dilated, and a tendency to sleep was noticed in every case; the throat was dry. The hallucinations, delirium, nausea and trembling which have been described by Wood and other observers as following the administration of the hydrobromate and hydriodate of hyoscine, were not observed by Sohrt and Kobert.

Hyoscine, according to Kobert, even in large doses, is well borne by some of the lower animals; a cat recovered after nine grains, and a small rabbit was unaffected by three grains. After large doses it is excreted unchanged by the kidneys. It influences but little the cerebral functions of the lower animals, but has a markedly calming effect on man when the brain is excited. The depression of the reflex excitability of the spinal cord, which, according to Wood, was present in lower animals after the administration of the hydrobromate, was not observed in the experiments made by Sohrt.

Claussen, who published his investigations on the action of hyoscine in 1883, stated that this drug stimulates the vagus and decreases the heart's frequency. Wood concluded that it had no influence on the vagus. Kobert, however, is of opinion that it paralyzes the vagus terminations in the lower animals, and probably also in man. In a lunatic with depressed circulation, which he attributed to inhibition exerted by the brain on the heart, he found that hyoscine always improved the pulse. In healthy men, however, increased rapidity of the pulse, which ought to follow vagus paralysis, was not always noticed. It antagonizes the influence of muscarin on the heart. In cold and warm blooded animals it dilates the peripheral vessels, probably by its paralyzing influence on the ganglia contained in their walls. Kobert does not think that it influences the vaso-motor centre, as the experiments of Wood led him to believe. The respiration is unaffected, according to Kobert, while Wood considers that this drug has a depressing influence on the respiratory functions, acting in all probability on the respiratory centres.

Hyoscine certainly diminishes the secretion of saliva, hence the drying of the throat which follows its use. The secretion of perspiration, too, is lessened, and some of Sohrt's patients who tried both hyoscine and atropine to check sweating preferred the former drug.

It is well known that muscarin increases intestinal peristalsis; hyoscine antagonizes the influence, paralyzing therefore the nerve apparatus on which the muscarin acts.

According to Dr. Emmert of Berne, hyoscine acts more energetically and rapidly as a mydriatic than either atropine or duboisin, and is effective even in solution of 1 part to 1000. Kobert, however, states that complete dilatation of the pupil is only produced by full doses. It acts, he says, by paralyzing the periphery of the oculo-motor nerve. Sohrt found that 1-140 of a grain dilated his pupils without paralyzing accommodation. Sohrt gave subcutaneously from 1-70 to 1-140 of a grain to a number of lunatics suffering from mental excitement and insomnia, who had taken other calmatives in vain. He administered the drug about one hundred times. Sleep was always produced, and no threatening symptoms were noticed.

As a rule, the patients fell asleep in ten to fifteen minutes after the injections, and did not awake for five to eight hours. A little fullness of the head was at times complained of during the morning following an injection. It is specially worthy of remark that the hyoscine produced good results in several cases in which hyoscyamine in full doses have been ineffectual. Kobert is inclined to consider hyoscine beneficial as a sedative in mental diseases only. —*Arch. f. exp. Path. und Pharm. XXII., Hft. 6.*

